# Implementation and scalability of a digital intervention to reduce depressive symptoms in people with diabetes, hypertension or both in Brazil and Peru: a qualitative study of health system’s stakeholders’ perspectives

**DOI:** 10.1007/s44192-022-00015-0

**Published:** 2022-06-03

**Authors:** V. Cavero, M. Toyama, H. Castro, M. T. Couto, L. Brandt, J. Quayle, P. R. Menezes, D. C. Mohr, R. Araya, J. J. Miranda, F. Diez-Canseco

**Affiliations:** 1 CRONICAS Center of Excellence in Chronic Diseases, Universidad Peruana Cayetano Heredia, Lima, Peru; 2 Population Mental Health Research Centre, Universidade de São Paulo, São Paulo, Brazil; 3 Department of Preventive Medicine, Faculdade de Medicina, Universidade de São Paulo, São Paulo, Brazil; 4 Center for Behavioral Intervention Technologies, Department of Preventive Medicine, Feinberg School of Medicine, Northwestern University, Chicago, IL USA; 5 Centre for Global Mental Health, Institute of Psychiatry, Psychology, and Neuroscience, King’s College London, London, UK; 6 Department of Medicine, School of Medicine, Universidad Peruana Cayetano Heredia, Lima, Peru

**Keywords:** Digital mental health, Qualitative research, Depression, Health services, Latin America, Implementation science

## Abstract

Two randomized controlled trials (RCTs) in Brazil and Peru demonstrated the effectiveness of CONEMO, a digital intervention supported by trained nurses or nurse assistants (NAs), to reduce depressive symptoms in people with diabetes and/or hypertension. This paper extends the RCTs findings by reflecting on the conditions needed for its wider implementation in routine care services. A qualitative study using semi-structured interviews and content analysis was conducted with nurses/NAs, clinicians, healthcare administrators, and policymakers. Informants reported that CONEMO would be feasible to implement in their health services, but some conditions could be improved before its scale-up: reducing workloads of healthcare workers; raising mental health awareness among clinicians and administrators; being able to inform, deliver and accompany the intervention; assuring appropriate training and supervision of nurses/NAs; and supporting the use of technology in public health services and by patients, especially older ones. We discuss some suggestions on how to overcome these challenges.

## introduction

Depression is the leading cause of disability and a highly prevalent condition worldwide [1]. It is more prevalent in certain groups, including people with non-communicable diseases (NCDs), such as diabetes [2] and cardiovascular diseases [3]. This comorbidity has been associated with worse health outcomes and quality of life, and with higher disability, morbidity and mortality [4]. A major challenge to address this issue is the scarcity of specialized care, leading to a large treatment gap, especially in low- and middle-income countries (LMICs). Two cost-effective strategies to close this gap are the use of technology to expand coverage, and the use of task-shifting, i.e. the delegation of specific tasks from one provider to another with shorter training and fewer qualifications, such as general doctors to nurses, or nurses to community health workers [5].

Aligned with these strategies and to address the high comorbidity between depression and NCDs [6, 7], the Latin America Treatment and Innovation Network for Mental Health (LATIN-MH) developed CONEMO, which stands for emotional control in Portuguese and Spanish and is a digital intervention to reduce depressive symptoms among people with diabetes, hypertension or both, supported by trained nurses or nurse assistants (NAs). The CONEMO intervention proved to be feasible and acceptable in pilot studies in São Paulo, Brazil, and Lima, Peru [8, 9]. Then, the effectiveness of CONEMO was demonstrated in two randomized control trials (RCTs), conducted between 2016 and 2018, in public primary and secondary healthcare clinics from São Paulo and Lima [10]. The intervention was effective in reducing depressive symptoms, in at least 50%, after three months compared with enhanced usual care in both countries [10].

These promising results encourage its implementation in Peru, Brazil and other resource constrained settings. However, many interventions successfully tested in RCTs are not further implemented in real-world settings due to a variety of factors, including healthcare policies, available funding or providers’ attitudes towards the intervention [11]. Likewise, interventions using task-shifting, such as CONEMO, are usually not expanded due to prioritization of psychiatrists’ care over other disciplines and biomedical approaches [12]. Thereby, retrieving feedback from key stakeholders, such as health workers or policymakers, before scaling-up effective interventions, allows the identification of such potential challenges [13], much needed before embarking on scaling up efforts. Since CONEMO was tested in routine practices, unlike most depression interventions [14], we had the opportunity to identify potential challenges from the point of view of key stakeholders from both countries who were involved in the implementation of the RCTs. Therefore, this study aims to describe and analyze the perceptions and experiences of different stakeholders about the implementation and potential scalability of CONEMO in public health services of Brazil and Peru.

## Methods

### Settings

In São Paulo, Brazil, a cluster RCT was conducted in 20 public Family Health Units (FHUs), involving their own staff of NAs in the intervention delivery. FHUs have a general healthcare administrator, and four to eight teams, which are composed of one medical doctor, a graduate nurse, two assistant nurses and five to ten community health workers. In Lima, Peru, an individual-level RCT was conducted in three public hospitals and four primary healthcare centers that deliver care to individuals presenting with hypertension or diabetes, with the support of three nurses hired by the research team, all with previous experience working in public health services. A broader description of the healthcare systems of Brazil [15] and Peru [16] has been reported elsewhere.

### The CONEMO intervention

CONEMO is a 6-week low-intensity psycho-educational intervention to reduce depressive symptoms in people with diabetes, hypertension or both. It is a digital mental health intervention, delivered through a mobile application (app), and supported by a nurse/NA. Based on the principles of behavioral activation [17], CONEMO comprises 18 sessions, three per week, that encourages the patient to perform pleasant activities (e.g. meeting a friend), healthy activities (e.g. take their medicines) and everyday tasks (e.g. pay the bills).

All patients in the intervention arm had an initial face-to-face appointment with a nurse/NA, where they received a loaned smartphone with the CONEMO app installed and were trained on the use of the device and the app. Nurses/NAs had two scheduled phone calls to monitor patients’ adherence to the sessions and solve difficulties with the technology, and extra phone calls if patients had low adherence to the intervention or requested help. Nurses/NAs used a tablet to access a dashboard to track their patients’ progress and register their tasks (e.g., calls conducted). If a patient was deemed at risk by the nurse (e.g., suicide risk), they were assessed by the research team and referred to a specialist, or a relative was contacted, or both. After the six-week period, patients met with a nurse/NA to return the smartphone.

In Brazil, the 440 patients of the intervention group were monitored by 55 NAs from the FHUs, with an average of 8 patients per NA. In Peru, the 217 patients in the intervention group were divided among three nurses hired full-time, who had between 72 and 74 patients each along the 9 months of the study [18]. The decision to hire nurses rather than using existing healthcare staff was based on the pilot studies conducted in Lima, which revealed a significant workload for staff nurses and challenges to perform the expected tasks [8, 9]. Nurses in Peru had previous experience using technology and received a longer training than in Brazil (40 vs 8 h respectively), which eased their learning of CONEMO [18]. Once a week, nurses/NAs in both countries had meetings with a clinical supervisor, a psychologist, to discuss patients’ adherence to CONEMO and solve any problems.

The RCTs’ methodologies and results [10] as well as the experience of the nurses/NAs’ training and supervision have been described in previous publications [18].

### Informants

Four groups of stakeholders of the public healthcare systems in Brazil and Peru were interviewed: (1) Nurses/NAs involved in the delivery of the CONEMO intervention during the RCTs; (2) Clinicians (e.g. physicians, nurses) who collaborated on the patients’ recruitment and referrals to specialized care when patients were deemed at risk; (3) Healthcare administrators of the health facilities who were periodically informed about the implementation of the study; and (4) Policymakers (e.g. government representatives of the Mental Health, NCDs, or FHUs Directorates at the Ministry of Health). Interviews were conducted by the RCT fieldwork coordinators in both countries, one RCT fieldwork supervisor in Peru, and two research assistants in Brazil. Research assistants received training to conduct the qualitative interviews by members of the research team who had large experience in qualitative methodology, whereas the RCT fieldwork coordinators and supervisor had vast experience conducting semi-structured interviews.

We used purposive sampling [19] to select the informants who were more knowledgeable about CONEMO and its implementation during the RCTs. In Brazil, one NA delivering the intervention from each FHU was interviewed. NAs were selected based on recommendations from the RCT’s fieldwork supervisors about NAs who expressed both positive and negative opinions about the intervention during the trial. In Peru, the three hired nurses were interviewed. Among the other stakeholders, we interviewed those who were more involved in the implementation of CONEMO RCTs or had roles within the healthcare systems that could be relevant for a future scale-up. We used the saturation theory to determine the final sample [20], which enabled us to reach a diverse range of experiences with the RCT process.

### Data collection tools

Semi-structured interview guides, one for each of the four informant profiles, were developed based on topics proposed in a research project shared by the US National Institute of Mental Health (NIMH) Collaborative Hubs [21], one of which was The Latin America Treatment and Innovation Network in Mental Health (LATIN-MH) project. These guides were then adapted by our team to make them culturally appropriate and relevant to the CONEMO intervention. Examples of these questions include: “How was your experience participating in this study?” and “Thinking about CONEMO, ¿which barriers do you identify for the implementation of this intervention in the public health system?” The topics included are presented in Table 1.

**Table 1:** Topics included in the interview guides

Informants’ profile	Topics
Nurses/NAs	Overall experience with the RCT
	Experience with their training
	Relationship with the patients
	Monitoring of patients
	Experience with the use of technology
	Perceived contextual factors of patients that may have influenced their participation in the study
	Facilitators and barriers for the scaling-up of CONEMO
Clinicians and healthcare administrators	Experience with the RCT
	Opinion about the use of technology and task-shifting
	Facilitators and barriers for the scaling-up of CONEMO
Policymakers	Opinion about the use of technology and task-shifting
	Facilitators and barriers for the scaling-up of CONEMO

### Procedures

Interviews were conducted from August to December 2018, just after the culmination of the RCTs follow-ups but before the data analysis was completed. At that point, informants did not know that the intervention proved to be effective. Informants in both countries were contacted by phone or in person and received a detailed explanation about the purpose of this qualitative study. Those interested in taking part scheduled a date to be interviewed. Interviews were conducted in person, recorded using a portable voice recorder, and subsequently transcribed verbatim. The average duration of the interviews in Brazil and Peru was 31 and 47 min, respectively.

### Data analysis

A content analysis of the interviews [22] was conducted using NVivo 12 software. This analysis involved the decontextualization stage [22], in which the research teams in both countries, comprised by those who conducted the interviews, designed a common coding list (Appendix 1) for each informant profile based on the interview guides, and then emerging codes were subsequently discussed and added. Once all data was coded, the research team recontextualized the findings, holding group discussions and identifying the most relevant information, particularly referring to the experience of interviewees with the intervention implementation and its potential scale-up. These codes were later condensed into broader categories. All of these categories were standardized among members of the research team across and within countries. Discrepancies among coders were discussed and solved together. During the analysis, the information gathered from different profiles were triangulated and synthesized [22], as shown in the results.

### Ethical considerations

The study protocols were approved by the NIMH Data and Safety Monitoring Board and local Ethics Committees in Brazil and Peru. Participation was voluntary and all informants signed an informed consent form before their involvement in the study. One author has accepted honoraria and consulting fees from Otsuka Pharmaceuticals, Optum Behavioral Health, Centerstone Research Institute, and the One Mind Foundation, royalties from Oxford Press, and has an ownership interest in Adaptive Health, Inc. Other authors report no known conflicts of interest. All authors certify their responsibility for the manuscript.

## Results

In total, 45 informants were interviewed, 26 in Brazil and 19 in Peru (Table 2). Most interviewees were women (77% in Brazil, 79% in Peru) with a mean age in both countries of 42 (SD 10.8). All were older than 18 and worked at one of the health clinics involved in the RCTs, except for the policymakers and Peruvian nurses.

**Table 2:** Number and type of informants per country

Informants	Brazil	Peru	Total
Nurses/NAs	10	3	13
Clinicians	9	10	19
Healthcare administrators	4	4	8
Policymakers	3	2	5
Total	26	19	45

The analysis of these interviews was organized into two main categories: “Perceptions and experiences about the implementation of CONEMO”, and “Potential challenges to scale-up the CONEMO intervention, and how to address them”. Both categories were divided into three subcategories, as shown in Table 3.

**Table 3:** Categories found after the qualitative content analysis of the study

Categories
**Perceptions and experiences about the implementation of CONEMO**
Informants’ opinions about using task-shifting in public health services
Nurses/NAs’ perceptions about their training and use of technology
Nurses/NAs’ experience with patients’ training and monitoring
**Potential challenges to scale-up the CONEMO intervention and how to address them**
*Challenges at the health-system level*
Lack of infrastructure and resources
Poor prioritization of mental health
Lack of use of technology within public health services
*Challenges at the provider level*
Nurses/NAs’ willingness and capacity to deliver CONEMO
Provision of mental health care to severe cases
*Challenges at the patient level*
Socioeconomic barriers to use and adhere to CONEMO
Patients’ difficulties with technology

### Perceptions and experiences about the implementation of CONEMO

This section summarizes the opinions of clinicians, healthcare administrators and policymakers of using nurses/NAs from public health services to support the intervention delivery. Then, it describes the nurses/NAs’ perceptions and experiences about their own training, their use of technology and the completion of their tasks.

#### Informants’ opinions about using task-shifting in public health services

Interviewees in Peru were well-informed and generally in favor of the use of task-shifting as a strategy to overcome the scarcity of specialists, not only in mental health, within public health services. Clinicians mentioned that task-shifting would expand the access to healthcare, enable collaboration among different professionals, and build over the existing close bonds between nurses and patients.

"I think [task-shifting] is a good idea because the closest health worker to the patient is a nurse (...) For us [doctors] it could be up to three months before we see a patient, but nurses see them every month or every time they require it (...), so to me it would be good to use staff closer to the patient" (Clinician-Family doctor, Peru, 27QHP02).

Healthcare administrators and policymakers were also in favor because of the lack of specialists and the ease to hire and train nurses instead of more expensive and scarce mental health professionals. Importantly, most informants emphasized the necessity of training and supervision when implementing task-shifting.

"I think it is good because we are in the innovation trend, delegating tasks to other groups. As long as there is training, I do not think it would be difficult for a nurse to do this" (Clinician-Nurse, Peru, 27QHP01).

In Brazil, however, most interviewees were not familiar with the concept of “task-shifting”, and even after explaining its meaning, some of them confused it with the technique of role playing whereas others explicitly said that they did not know about this strategy.

#### Nurses/NAs’ perceptions about their training and use of technology

In Brazil, NAs highlighted the importance of the training received to learn how to use the tablet; but found it too short, suggesting a longer session or some refreshers. They said that they learned while doing, helping each other, or receiving help from the project clinical supervisor. In contrast, Peruvian nurses had a longer training, which they considered to be sufficient and did not find major difficulties.

"If something remained unclear after the training, then when [the supervisor] came, she was able to help and I thought this was very good, because the training was very short" (NA, Brazil, QAE03)

Nurses/NAs in both countries found the tablet (device) and the dashboard (program) easy to use. Brazilian NAs reported some difficulties using the dashboard at the beginning, but they also mentioned that using it daily made it easier over time.

#### Nurses/NAs’ experience with patients’ training and monitoring

Nurses/NAs trained their patients on the use of the smartphone and the app. In both sites, they experienced more difficulties with older patients. Peruvian nurses mentioned that the training was longer with these patients, and some were nervous about using the phone; whereas in Brazil, NAs mentioned that many patients forgot to charge their phone and thereby were unable to use the app.

Regarding the monitoring calls, one nurse in Peru mentioned that, at the beginning, when she did not have much experience with the smartphone and the app, it was difficult to understand the patients’ doubts because they were not clear on their queries, but as she became more familiar with the technology, it was easier to help them.

Nurses/NAs in both countries perceived that most patients were satisfied with the monitoring calls. Some patients seemed to enjoy being called and looking forward to receiving the next call; while a few were more reluctant to answer the phone.

“Some were very surprised [with the call], because they weren't doing anything—“I forgot about it…", they would say. They weren't doing anything. Others liked it, some asked me: “Are you going to call again tomorrow?” So, we noticed there was a need [of someone caring about them]. “How often will you call me? Oh, you take too long to call back”. So, that’s it, they thought we had to call them all the time, sometimes they even called at the clinic: "Oh, can I talk to the lady of CONEMO?" (NA, Brazil, QAE06)

“They felt good that someone else started calling them to ask how they were and if they had any difficulties with CONEMO (…) They were always grateful [with the calls], because they said that CONEMO was like their companion, as if CONEMO was a companion so that they did not feel alone”. (Nurse, Peru, QNI01)

Other problems mentioned by the nurses/NAs were difficulties in scheduling in-person meetings, due to patients not answering the phone, incorrect phone numbers, patients arriving early, late, or missing the appointments.

In Brazil, many NAs felt overwhelmed by the amount of work and could not conduct all their CONEMO duties on time. On the contrary, the three Peruvian nurses stated that the workload was good, since they did not have conflicting tasks and were exclusively accompanying CONEMO. Since the allocation of patients was progressive and the appointments were arranged by nurses and patients, they were able to organize themselves without feeling overwhelmed.

### Potential challenges to scale-up the CONEMO intervention and how to address them

Informants in both countries deemed CONEMO to be a positive intervention to be provided in public health services, and suitable to be implemented in their health systems. They were in favor of using technology within public health services, working with non-specialized mental health providers to have a more efficient use of the scarce resources, having a structured but flexible program to treat depression, or expanding the access to mental healthcare.

“I find it interesting [that CONEMO] does not depend on the services, of the professional (…) also that it offers a flexible structure, like a therapeutic plan, out of the health centers (…) I find it very interesting to include technology because we have a great demand [from patients] and we require affordable interventions, that are accessible and that are accepted by the population” (Policymaker, Peru, 00QPM01)

“I think that implementing it [CONEMO] as a public policy would be of great value, because it strengthens and reinforces what we already do, right? And I think it has the potential to address things that can go unnoticed, such as vulnerabilities or risk situations, where normally, access is not totally appropriate." (Policymaker, Brazil, QPM02)

Yet, informants in both countries were well-informed about their public health systems’ conditions and identified some challenges for scaling up CONEMO. Here, we present them organized at the health system-, provider-, and patient-level, together with the existing and potential conditions that could ease the implementation of CONEMO mentioned by the interviewees.

#### Challenges at the health-system level

##### Lack of infrastructure and resources

In both countries, most informants identified some challenges to potentially scale-up CONEMO within their public health system. These included the heterogeneity of health services (i.e., some able to implement the intervention while others not because of different numbers of patients, personnel, available space, etc.), poor coordination with mental health services to ease the referral of patients, lack of space for in-person meetings, scarce human resources to accompany CONEMO, and low budget.

“First, it will depend on, as they say, the budget, the money, and the human resources. We do not have enough to cope with so many patients and [we need] space too. Look where we are working, it is a very small office for the whole Endocrinology service” (Clinician-Endocrinologist, Peru, 23QHP02).

In Peru, one clinician highlighted that the Ministry of Health had a specific budget for mental health, which could be used to buy electronic devices to use CONEMO, but policymakers may not want to use it for that purpose.

“I don't know if the government will have to invest in mobile devices, but they can certainly do it, because mental health has budget, but some do not want to use it" (Clinician-Psychologist, Peru, 23QHP03).

A Peruvian clinician mentioned that health centers have their patients’ contact information, which would help them reach the patients who could benefit from CONEMO. Informants also mentioned that the lack of space and scarce human resources could be addressed by offering CONEMO within services that treat people with NCDs, such as those in which the trials were implemented (e.g., Endocrinology services).

##### Poor prioritization of mental health

Many informants in both countries shared the idea that mental health and treating people with mental disorders were not priorities for health authorities.

“The authorities, the ministers, they are the problem, they do not give importance to these cases" (Clinician-Endocrinologist, Peru, 23QHP02).

Informants mentioned that some strategies to raise awareness and commit policymakers on the scaling-up of CONEMO would be the dissemination of the RCTs’ results if found positive, showing its benefits in terms of cost savings and recovery of patients, and the possibility to have better trained personnel. Additionally, they recommended engaging the Ministry of Health’s authorities to establish a performance indicator for CONEMO. Thus, healthcare administrators would be required to meet such an indicator, ensuring its implementation and continuity.

“First of all, to let authorities know, how much benefit is obtained (...) because most authorities use indicators expressed in numbers and production rather than quality and recovery, so we have to make them understand the benefit of recovery and the reductions of costs in complications” (Healthcare administrator, Peru, 24QHM01).

##### Lack of use of technology within public health services

Some interviewees mentioned that their health systems do not use much technology because they do not have the equipment, internet connection and proper training, which could be challenging for scaling-up CONEMO. Policymakers were asked if they knew of current initiatives using technology. In Brazil, one of them shared two initiatives, one in which community health workers used tablets and smartphones to support their work, which speeds the communication with their teams; and another in which health professionals share and discuss their cases through chat groups in WhatsApp. Policymakers in both countries said that there were plans to use electronic health records in public health services, but services were still using paper records.

In Peru, a policymaker said that obstetrics and neonatology services use telemedicine, but in mental health services it was only rarely used. Another Peruvian policymaker mentioned virtual training, but also commented that there was still a long road to go for a wider use of technology in the health system, especially in terms of the lack of electronic health records.

#### Challenges at the provider level

##### Nurses/NAs’ willingness and capacity to deliver CONEMO

Most informants raised different concerns about public health system’s nurses/NAs being able to incorporate CONEMO in their routines. Firstly, non-specialists do not prioritize mental health care. Administrators and clinicians stated that actively involving frontline teams when designing a new intervention would gain their buy-in. Importantly, some clinicians mentioned that health workers were aware of the importance of mental health and motivated to provide good care; and if informed about the effectiveness of CONEMO, they could be more engaged.

“Sometimes we see a super cool program, super cool, because on paper everything looks great. But then, when you see it implemented, the implementation is a bit difficult. So, I believe that by engaging front-line workers, the program becomes more effective.” (Healthcare administrator, Brazil, QGR03)

Secondly, most informants in both countries said that nurses/NAs might have difficulties to implement CONEMO because of high workloads and limited time to be trained, refer, and monitor their patients.

“An impediment, I believe, would be the availability of nursing assistants in the case of a very large group of patients. A small group, we manage to handle it once a week, an hour. But a larger group, we would not be able to handle.” (Healthcare administrator, Brazil, QGR02)

To address this, informants suggested reorganizing nurses/NAs’ tasks to incorporate the training and monitoring into their routines, expanding their consultation time, providing monetary incentives, hiring new personnel to support the intervention, or implementing it in other settings, such as community mental health centers, which are small but numerous centers at the primary care level that provide specialized mental healthcare.

Thirdly, policymakers and clinicians emphasized the need to train those delivering the intervention to assure good-quality care. One Peruvian nurse suggested informing the whole workforce –not only nurses and psychologists– about CONEMO to increase the referral of patients to use it.

##### Provision of mental health care to severe cases

One Peruvian mental health clinician said that due to her high workload, she was not able to attend all patients who required specialized care during the trial. She tried to contact them later, but some could not be reached by phone. In Brazil, a clinician mentioned that referrals to specialized care were decided on the basis of a patient's socioeconomic status, since it was feared that due to the lack of financial conditions, patients would not attend.

#### Challenges at the patient level

##### Socioeconomic barriers to use and adhere to CONEMO

This group of barriers include difficulties with transportation and having a permanent cellphone. Regarding transportation, some healthcare administrators and clinicians mentioned that patients may have difficulties attending the health center for the training session. A clinician related this to economic constraints, because going to the health center would mean missing a workday. This lack of resources could also limit patients’ capacity to own a cellphone or have internet access. Additionally, one Peruvian policymaker said that many patients change their phone numbers frequently, affecting their continuity of care and the monitoring of CONEMO.

"We are in an area that does not have economic power (...) Then asking a patient to have a cellphone with enough mobile data to use a program like this (...) especially, if they are going to use videos, would be complicated" (Clinician-Endocrinologist, Peru, 21QHP02).

##### Patients’ difficulties with technology

Informants in Peru and Brazil said that some patients, especially the older ones, may not know how to use a cellphone, have physical limitations to use it (visual, hearing or motor impairments), low literacy, little practice, and lack of support from the family for dealing with technology. For instance, a Peruvian interviewee said that in a program for diabetes that used SMS reminders, the cellphone was shared by different family members, so reminders were not going to the person who required them. One healthcare administrator recommended engaging patients’ families to the CONEMO intervention to encourage its use and solve difficulties, which could also improve the communication of patients with their families.

“[You should] approach the family to make them understand what you are doing and help [the patients]. Generally, people with hypertension, diabetes, are much older, and older people are isolated, most of the time, because their children go away, logically. They have less mobility, go out less, so they are a bit isolated, so the cellphone brings them closer, closer to the world, and if there is someone who communicates with them, they will be more willing to provide support” (Healthcare administrator, Peru, 24QHM01)

Importantly, despite initial doubts about patients’ ability to use the app, nurses/NAs in both countries mentioned that most patients were able to use it and did not face as many difficulties as they anticipated.

"[CONEMO] seemed to be kind of difficult at first. I even wondered: would a patient be able to use this? But then, I accompanied them, I saw that they learned how to use it, and, in the end, it was nothing like I thought. It was so easy that anyone could do it." (Nurse, QAE01, Brazil).

## Discussion

This qualitative study aimed to describe and analyze the perceptions and experiences of the nurses/NAs delivering the CONEMO intervention, as well as clinicians, healthcare administrators, and policymakers from the public health systems of Brazil and Peru about the implementation of CONEMO, in addition to their perceptions about the most relevant challenges and potential solutions to its future scale-up. Based on their experience with the RCTs and their knowledge of how their healthcare systems work, informants found that CONEMO would be feasible to implement in public health services of Brazil and Peru. This aligns with previous studies that showed that CONEMO was effective and acceptable to use by public health services’ patients with diabetes, hypertension, or both [10, 23].

For the scalability of CONEMO, most stakeholders, including the nurses/NAs involved in the trial, discussed human resources availability to support this intervention in routine practices due to heavy workloads. In our study, Brazilian nurses/NAs faced higher constraints than their counterparts in Peru –who were exclusively hired to support the intervention– to complete CONEMO activities on time. Indeed, meetings of Brazilian nurses/NAs with their clinical supervisor were usually used to complete their CONEMO tasks due to difficulties to conduct them during their working hours [18]. This is one of the biggest and most common challenges when implementing new interventions in scarce-resource settings [24] and has been previously reported in Peru [25] and Brazil [26]. Strategies to reduce this overburden include the allocation of more funding, incentives for the workforce –beyond monetary incentives only– [27], standardizing and simplifying providers’ tasks, or involving other providers, such as community health workers [28]. Our interviewees also recommended reorganizing nurses/NAs tasks and introducing CONEMO in non-mental health services (e.g., cardiology services), which others have also suggested [4, 29]. The implementation in Peru also showed that nurses specifically hired to support CONEMO could significantly reduce this burden and deal with a larger number of patients.

Interviewees also reflected on how to improve the buy-in from stakeholders at different care levels for mental health interventions. Proposed suggestions include sensitizing clinicians to motivate patients to use CONEMO, like a prescription; engage policymakers to introduce a performance indicator for CONEMO, so that healthcare administrators may be encouraged to improve the working conditions of nurses/NAs and to provide more and better support for the intervention; and showing policymakers the positive results from the RCTs. The latter recommendation aligns with suggestions made in other LMICs about the information and clear guidance that policymakers usually lack about effective interventions [30], and that CONEMO could address by showing policymakers that this is an effective intervention to treat depression, as shown in its successfully implemented trials. This is a key role that researchers can play to enhance the learning processes of health systems and thereby the care provided in public health services [31]. Indeed, the results of CONEMO in terms of reduction of depressive symptoms as well as improvement in quality of life and functioning [10], could be used as an indicator of high-quality health systems [32].

Importantly, the low prioritization of mental health found in our study might have changed in the context of the COVID-19 pandemic. Policymakers may be more aware of the mental health needs of their population, as exemplified by the promptly published Peruvian mental health plan during the first months of the pandemic which highlights the necessity to attend to the mental health impact of COVID-19 [33] and the use of technology within public health services [34, 35]. This improved prioritization is also aligned with the progress made in Peru and Brazil to enhance the mental health of their populations, integrate mental health care into other health programs, and expand the provision of community-based mental health services [36–38].

Regarding the specific component of task-shifting of CONEMO, we found that even though most stakeholders were positive about using it in health services, they emphasized the need to assure good training and supervision for non-specialists to perform their tasks, as recommended when implementing this strategy [39], something that was paramount during the RCTs [18]. Interviewed nurses/NAs also stated the importance of their training and constant supervision, but the ones from Brazil emphasized the need for longer training and refreshment sessions. In this regard, other LMICs have tested trainings with long periods of practice for non-specialists providing mental healthcare, finding positive results in knowledge, attitude and self-efficacy [40]. Importantly, although our informants did not mention any concerns about “replacing” specialists’ jobs, this would be relevant to consider since it is a common difficulty when implementing task-shifting strategies [41].

Finally, the technology component of the intervention involved some challenges. Stakeholders in both countries mentioned that digital interventions were not widely used in their health services. This scenario might have changed due to the global expansion of telemedicine in public health services brought about by the COVID-19 pandemic, including the health systems of Peru and Brazil [42], and services directed to older populations [43]. This expansion could enable the integration of CONEMO in current non-mental health digital programs or incorporate other health interventions into the nurses/NAs’ CONEMO dashboard. Although technology literacy is still a relevant challenge in digital mental health, including in high income countries [44, 45], interviewed nurses/NAs reported that even their older patients were able to use and adhere to the intervention, which was also found in previous studies of CONEMO, in which even the elderly were able to use this digital intervention and complete all the scheduled sessions [10]. Another potential challenge related to technology is the need for patients to own a smartphone to access CONEMO. In the RCTs, patients received a loaned phone, but in a potential scaling-up, patients would need to have their own cellphone. This situation may have also changed along the years, especially after the COVID-19 pandemic, which has enabled a wider use of technologies in older adults, including those living in Latin American countries, to keep in contact with their relatives and friends during quarantines [46] and receive healthcare [43].

### Implications for policy

The implementation of CONEMO, a digital mental health intervention supported by nurses/NA, would be feasible in the public health services of Brazil and Peru; but some improvements suggested by our informants may ease its wider expansion. For instance, some policies could be implemented to reduce the high workloads of healthcare workers, such as having providers to specifically implement CONEMO, involving community health workers to support the intervention instead of nurses/NAs, or integrating CONEMO in other health services or community settings. It would also be important to inform the whole workforce about CONEMO, in order to ease their approach to their patients with simple and clear guidance on the intervention, which has shown positive results in improving patients’ engagement [47]. Likewise, it would be relevant to build communication channels to enhance the referral of patients to use CONEMO, as well as the referral to specialized mental health care when needed. Importantly, some pressing adaptations already implemented as part of enhancing the provision of healthcare services due to the COVID-19 pandemic can support the uptake of CONEMO, such as the wider expansion of telemedicine. In addition, since these RCTs were conducted, mental health has gained more importance among the public and policymakers, who designed policy guidelines to promote self-care and enhance the detection and treatment of psychosocial problems and mental disorders in public health services [35]. In this scenario, CONEMO could be a new and effective alternative that providers could offer to their patients.

## Strengths and limitations

A strength of this study is that it gathered and triangulated the perspectives of 45 stakeholders at different care levels of two countries, Brazil and Peru, who had a good understanding of the healthcare systems in which our intervention’ effectiveness was tested. This first-hand experience enabled them to identify potential pitfalls in the implementation and potential scaling-up of CONEMO, as well as pathways to enhance its uptake, and sustained implementation. Since this intervention proved to be effective to reduce depressive symptoms in both settings, this qualitative study findings are especially relevant to better understand the conditions needed to improve before pursuing a larger implementation.

One limitation is that most stakeholders, except for the nurses/NAs, did not have a detailed understanding of CONEMO, so that their inputs were not very much centered around the intervention’s principles or contents to improve patients’ mental health, but around the conditions and potential challenges to incorporate this intervention into their quotidian routines. These findings, however, could be informative for those who are considering implementing a digital mental health intervention supported by non-specialists into routine care services, in settings with poor infrastructure, overburdened providers, or low budget. Likewise, informants did not know, at that time, that the RCTs proved to be effective thus reducing some biases including social desirability bias. They reflected on the possibility of implementing CONEMO under the assumption that the intervention would be effective, so that the experiences we report here might not differ much if they would indeed have known the results.

## Conclusion

This study analyzed the viewpoints of different stakeholders about the implementation and scalability of CONEMO, a digital mental health intervention using task-shifting, in public health services of Brazil and Peru. Informants found that this intervention was feasible to implement and could be a promising option to be scaled-up in their public health systems. They also identified some conditions that could be improved and proposed some potential strategies in this regard. Challenges identified were the overburden of healthcare workers, poor prioritization of mental health among relevant stakeholders, the necessity to assure proper training and supervision of those supporting the intervention, and the lack of technology use in health services and by patients, especially by older people. However, some of these conditions may be less prominent nowadays since public health systems’ responses to the COVID-19 pandemic might have improved the prioritization of mental health care and the use of technology. Finally, even though Peru and Brazil have different healthcare systems, they both share similar challenges also present in several LMICs, such as scarce human, material and economic resources or high workloads, making these findings and potential solutions relevant to other resource constrained settings across LMICs and high-income settings.

## Data Availability

The datasets generated during and/or analysed during the current study are available from the corresponding author on reasonable request.

## Appendix 1: Codebook

**Table:**

Codes	Profile of the interview guides
1. Experience and opinion about the intervention components: Technology and task shifting	
1.1 Opinion about the task-shifting	Clinicians, healthcare administrators and policymakers
1.2 Problems with the Tablet	Nurses/NAs
1.3 Opinion of the Dashboard	Nurses/NAs
2. Training and monitoring of patients	
2.1 Difficulties with the training of patients	Nurses/NAs
2.2 Difficulties with the monitoring of patients	Nurses/NAs
2.3 Nurses/NAs’ workload	Nurses/NAs
3. Facilitators and barriers	
3.1 Facilitators to the implementation of CONEMO	Clinicians, nurses/NAs, healthcare administrators and policymakers
3.2 Barriers to the implementation of CONEMO	Clinicians, nurses/NAs, healthcare administrators and policymakers
3.3 Difficulties found during the RCT	Clinicians
3.4 Problems encountered when implementing new treatments in the health system	Clinicians, healthcare administrators and policymakers
3.5 Knowledge of new technologies implemented on the health system	Policymakers
